# Distinct online and offline effects of alpha and beta transcranial alternating current stimulation (tACS) on continuous bimanual performance and task-set switching

**DOI:** 10.1038/s41598-019-39900-0

**Published:** 2019-02-28

**Authors:** Kirstin-Friederike Heise, Thiago Santos Monteiro, Inge Leunissen, Dante Mantini, Stephan P. Swinnen

**Affiliations:** 1 Research Center for Movement Control and Neuroplasticity, Department of Movement Sciences, KU Leuven, Leuven, Belgium; 2 Leuven Brain Institute (LBI), KU Leuven, Leuven, Belgium; 3 Functional Neuroimaging Laboratory, IRCCS San Camillo Hospital, Venice, Italy

## Abstract

In the present study we examined the effect of bihemispheric in-phase synchronization of motor cortical rhythms on complex bimanual coordination. Twenty young healthy volunteers received 10 Hz or 20 Hz tACS in a double-blind crossover design while performing a bimanual task-set switching paradigm. We used a bilateral high-density montage centred over the hand knob representation within the primary motor cortices to apply tACS time-locked to the switching events. Online tACS in either frequency led to faster but more erroneous switching transitions compared to trials without active stimulation. When comparing stimulation frequencies, 10 Hz stimulation resulted in higher error rates and slower switching transitions than 20 Hz stimulation. Furthermore, the stimulation frequencies showed distinct carry-over effects in trials following stimulation trains. Non-stimulated switching transitions were generally faster but continuous performance became more erroneous over time in the 20 Hz condition. We suggest that the behavioural effects of bifocal in-phase tACS are explained by online synchronization of long-range interhemispheric sensorimotor oscillations, which impacts on interhemispheric information flow and the top-down control required for flexible control of complex bimanual actions. Different stimulation frequencies may lead to distinct offline effects, which potentially accumulate over time and therefore need to be taken into account when evaluating subsequent performance.

## Introduction

Transitions between different tasks are ubiquitous in everyday life and the ability to flexibly and fluently switch between different activities seems to become even more part of our contemporary multimedia reality. For switching between two different tasks one usually pays a price: responses following a switch are considerably slowed down and typically more susceptible to slips. But what is it that makes the switch between different tasks so difficult? The available body of literature suggests that the required reconfiguration of a task set potentially involves several processes that are essential for selection and implementation of the appropriate task set in a given situation: Identifying the new goal and shifting attention between specific features of stimuli or conceptual elements, updating working memory, inhibiting the preceding task set and activating the new task set, as well as monitoring the on-going performance^1^.

Albeit cognitive task-set switching paradigms are well established, task-set switching paradigms in the sensorimotor domain are vastly understudied^1^. Bimanual coordination allows investigating intentional task-set switching in the experimental setting by cueing participants to switch between different bimanual coordination patterns^2–6^. For the coordination and integration of motor actions of both hands, the information flow between the homologous primary motor and premotor areas has been attributed a prominent role [e.g. transcranial magnetic stimulation TMS^7–9^, positron emission tomography (PET)^10^, functional magnetic resonance imaging (fMRI)^11–13^, diffusion tensor imaging (DTI) and TMS^14^, electroencephalography (EEG)^15–18^]. While the control of congruent bimanual movements (such as mirror-image patterns) represents an intrinsically available motor programme, movements requiring spatially and/or temporally incongruent coordination of the two hands show lower stability and require more complex interhemispheric communication flow involving inhibitory mechanisms in order to prevent involuntary transition into a more stable coordination mode^19,20^. Within the sensorimotor network, these inhibitory control mechanisms are potentially conveyed through specific spectral content, in particular neural oscillations in the alpha- (8–12 Hz) and beta- (15–30 Hz) frequency range among local and between distant neural populations^21–25^.

On the one hand, local and inter-regional alpha band synchronization has been interpreted as a correlate for perceptual filtering function^26^ and a general inhibition of task-irrelevant regions^24,27,28^. On the other hand, more general inhibitory mechanisms and mechanisms controlling defined movement parameters (such as speed) have been attributed to the synchronization in the beta range^15,29,30^. Both, alpha and beta oscillations have been suggested to control the task-relevant selection and de-selection of sensorimotor neural ensembles, with alpha barring interference with movement selection and beta disinhibiting appropriate populations^31^ and assuring maintenance of the current state^25^. Consequently, it is conceivable that when these control mechanisms fail, transitions between different coordination patterns are allegedly hampered^32,33^. However, considering the controversial findings in this regard^15,18^, the question remains whether the respective behavioural relevance of interhemispheric synchronization in the alpha or beta spectrum and their specific behavioural relevance in the context of bimanual task-set switching can be differentiated.

To address this question we made use of transcranial Alternating Current Stimulation (tACS), which has shown the capacity to modulate the power of oscillatory rhythms in the brain in a frequency-dependent manner^34,35^ by synchronizing neural populations^36,37^ across distant brain regions^34,38,39^.

We applied concurrent bifocal tACS over left and right sensorimotor areas to externally modulate interhemispheric interactions. Our objective was to evaluate the behavioural effects of the potential in-phase synchronization of motor cortical neural activity to either the alpha (10 Hz) or beta (20 Hz) frequency during the performance of a bimanual task-set switching paradigm. This experimental implementation of bimanual task-set switching combines the transitions between two coordination modes with different levels of difficulty, i.e. a more stable mirror symmetric and a less stable parallel coordination pattern, and it has shown to elicit asymmetric switch costs^40–43^.

We hypothesized that bilateral modulation of alpha synchronization would primarily impact on the control of interference during switching transitions and hence would be mainly visible in the quality of performance, i.e. error rates. We furthermore hypothesized that its effects would be most pronounced in the more difficult transitions from the pattern of higher into the pattern of lower stability. Conversely, altered beta synchronization was hypothesized to mainly impact on the coordinative movement parameters and stability, and hence the overall speed of transitions potentially leading to movement slowing.

## Results

All 20 volunteers participated in both experimental sessions and a total of 408 trials (220 transitions, 146 continuation trials, 42 start/pause trials) were collected per participant and session. The dataset of one participant had to be excluded from further analyses because sufficient task familiarization could not be achieved, i.e. for this person the association between visual cues and response (coordination pattern) was not possible to reliably memorize and integrate within one familiarization and training session of >45 minutes (>2 SD above group mean). For the remaining 19 participants, familiarization and training lasted on average 12.82 ± 5.41 minutes. Familiarization and training duration was longer in the first session (15.53 ± 6.20 min.) than in the second session (10.11 ± 2.49 min., F(1,38) = 9.035, p < 0.01).

The amount of interhemispheric coupling has been shown to be dependent on the performance speed with higher speed requiring higher interhemispheric coupling^18,44^. Individual target frequency increased from the first (2.5 ± 0.4 Hz) to the second session (2.9 ± 0.4 Hz, F(1,38) = 10.02, p < 0.01). There was no systematic difference of training duration between 10 Hz and 20 Hz session (p > 0.9).

### Transcranial alternating current stimulation

For an exemplary participant, simulation revealed an electrical field with two circular foci centred around left and right primary and premotor cortices. Furthermore, electrical field simulation yielded an expansion of the field into deeper cortical layers as well as the corpus callosum (Fig. 2B).

**Figure 1:**
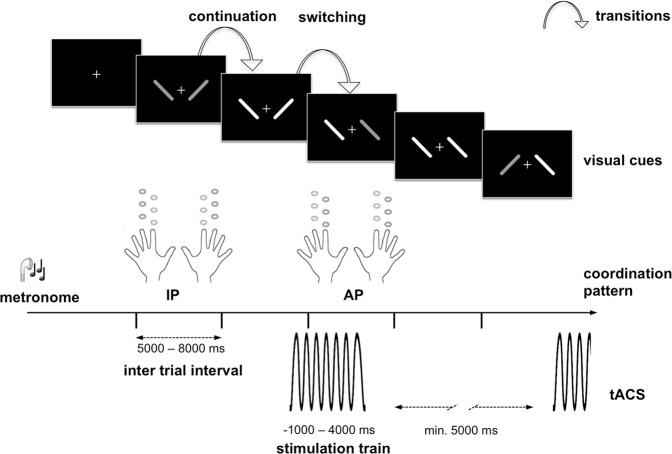
Experimental procedures. The bimanual motor paradigm with in- versus antiphase (IP, AP) bilateral tapping of index and middle fingers of both hands was performed on a custom-made keyboard. Individual tapping frequency was auditory paced and visual target cues indicated which movement pattern to perform. Event-related alternating current stimulation ensued randomly in 50% of the switching transition trials. Stimulation commenced 1000 ms before visual cue onset and lasted for a total of 5000 ms exclusive fading in/out phase of additional 500 ms. Between the end of the previous and the start of the next stimulation trains was a minimum interval of 5000 ms.

**Figure 2:**
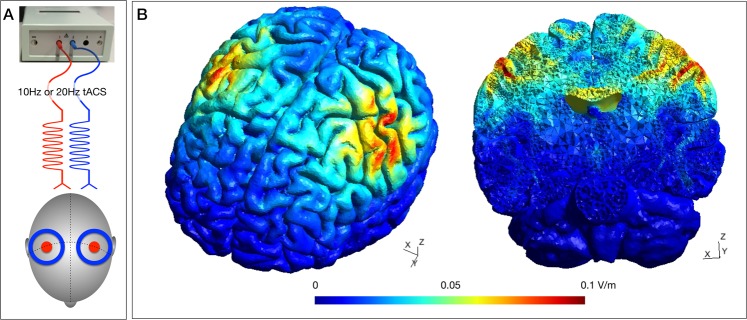
Transcranial alternating current stimulation. (**A**) Bifocal electrode montage with centre electrodes (surface area 9 cm^2^, diameter 3.4 cm) over C3 and C4 indicated in red, outer ring (surface area 35 cm^2^, inner diameter 7.5 cm, outer diameter 10 cm) indicated in blue. By using Y-connector cables, a 0° phase difference was achieved between centre electrodes (red) and between outer ring electrodes (blue). (**B**) Simulation of the electrical field of the bifocal centre-ring montage. Alpha (10 Hz) or beta (20 Hz) tACS was applied with a stimulation intensity of 1000 µA peak-to-peak, which resulted in a maximum electrical field strength of 0.1 mV/m with its foci over the primary sensorimotor regions of both hemispheres.

Impedance ranges varied slightly between the stimulation frequencies: while no difference was found for minimum impedance (10 Hz session 0.91 ± 0.80 kΩ, 20 Hz session 0.88 ± 0.59 kΩ), maximum impedance was slightly lower in the 20 Hz session (10 Hz session 4.41 ± 1.89 kΩ, 20 Hz session 4.225 ± 1.83 kΩ, F(1,18) = 13.91, p < 0.01).

### Subjective level of discomfort caused by tACS and self-perceived level of fatigue

All volunteers tolerated the stimulation and no adverse effects were reported. Analysis of variance of the discomfort rating (VAS_discomfort_) yielded overall very low ratings of subjective level of discomfort (average 0.86 ± 1.22) and no effect of STIMULATION CONDITION or SESSION was found (all p > 0.7). Among the most frequently reported expected neurosensory side effects were “tingling” (5 cases) or “pinching” skin sensations (3 cases). In 2 cases a “burning” sensation was reported. In two cases a mild skin irritation (transient reddening under the centre electrode) was observed.

Irrespective of SESSION or STIMULATION CONDITION (all p > 0.2), participants rated their level of fatigue significantly higher after the experimental session (VAS_fatigue_ pre: 2.34 ± 2.2, post: 4.64 ± 2.34, TIME POINT, F(1, 106.2) = 19.65, p < 0.0001).

### Behaviour during bimanual task-set transitions

**Figure 3:**
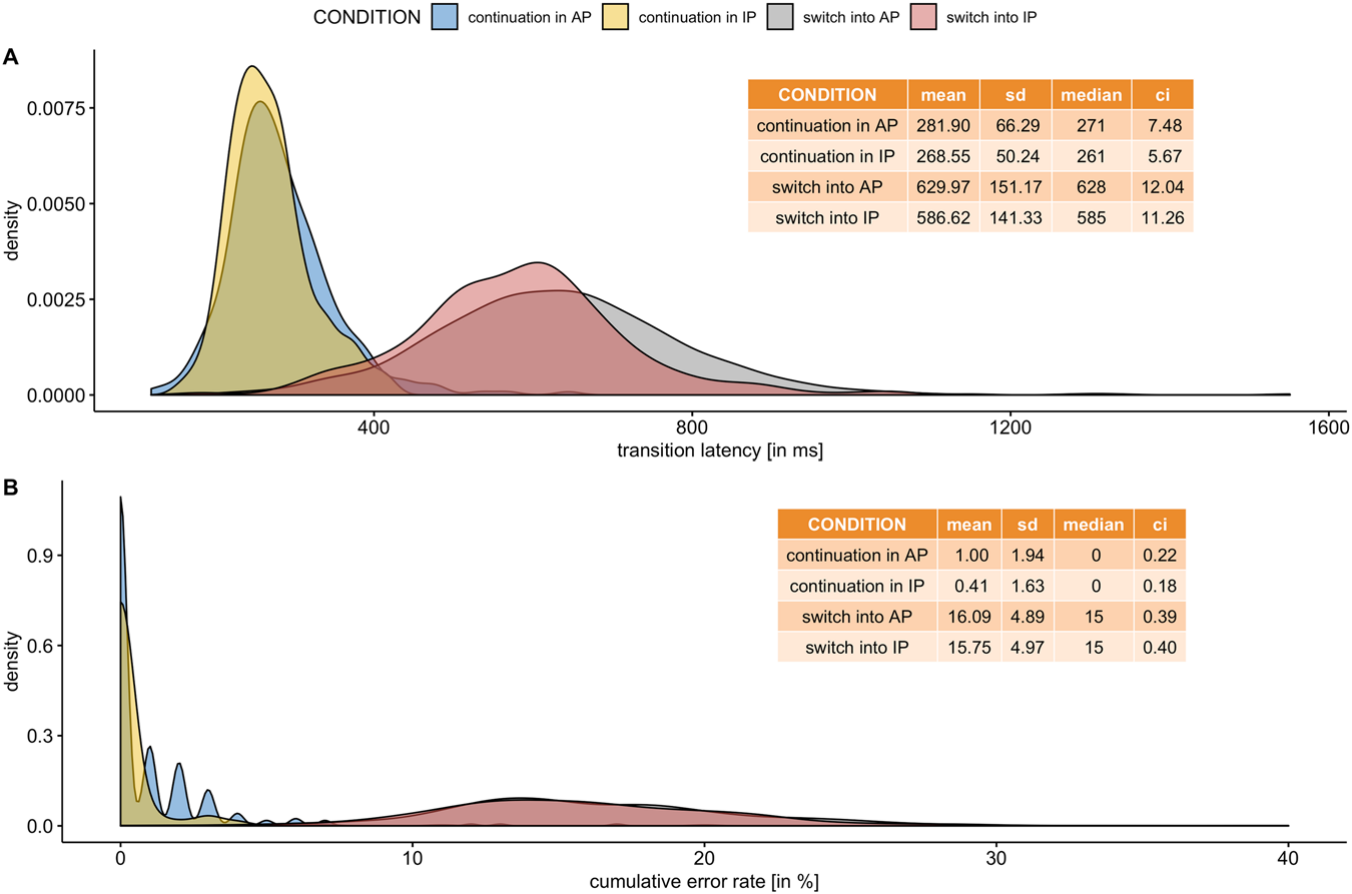
Density plots for (**A**) transition latency (in ms) and (**B**) cumulative error rate (in % of tapping/trial) and for the two transition conditions (continuation vs. switching) and coordination patterns (AP vs. IP). Individual performance conditions are coded in transparent colours (continuation in AP - blue, continuation in AP - yellow, switch into AP - red, switch into IP - grey). Descriptive statistics are summarized in the respective tables.

Overview of distribution and summary statistics of the three outcome parameters across conditions is depicted in Fig. 3.

### Multivariate Analysis of tACS effects on continuation transitions

The MANCOVA results (Type I sums of squares are reported throughout) revealed a significant main effect of COORDINATION PATTERN on performance in continuation trials (Pillai’s trace = 0.0419, *F*(2, 580) = 12.68, p < 0.0001) and a significant influence of tapping frequency (TARGET FREQUENCY, Pillai’s trace = 0.0925, *F*(2, 580) = 29.55, p < 0.0001) with higher target frequencies leading to considerably lower transition latencies and slightly higher error rates (overview of MANOVA results given in Table 1).

**Table 1:** MANCOVA results for continuation trials.

Multivariate Effects		Pillai’s Trace	Approx F (df, Error df)	Pr(>F)
COORDINATION PATTERN		0.0419	12.68 (2, 580)***	0.000004***
STIMULATION FREQUENCY		0.0015	0.42 (2, 580)	0.7
TIME centred		0.0061	1.77 (2, 580)	0.2
TARGET FREQUENCY centred		0.0925	29.55 (2, 580)***	0.0000000000006***
COORDINATION PATTERN*STIMULATION FREQUENCY		0.0004	0.11 (2, 580)	0.9
COORDINATION PATTERN*TIME centred		0.0025	0.73 (2, 580)	0.5
STIMULATION FREQUENCY*TIME centred		0.0162	4.78 (2, 580)**	0.009**
COORDINATION PATTERN*STIMULATION FREQUENCY*TIME centred		0.0030	0.87 (2, 580)	0.4
Residuals		581		
Residual standard errors		51.24		
		1.70		
Error: subject level	Pillai	approx *F* (*df*, Error *df*)		
TARGET FREQUENCY centred	0.6183	12.96 (2, 16)***		
Residuals	17			

Type I sums of squares given. Asterisks indicate level of significance at ***p < 0.0001, **p < 0.01, *p < 0.05.

**Figure 4:**
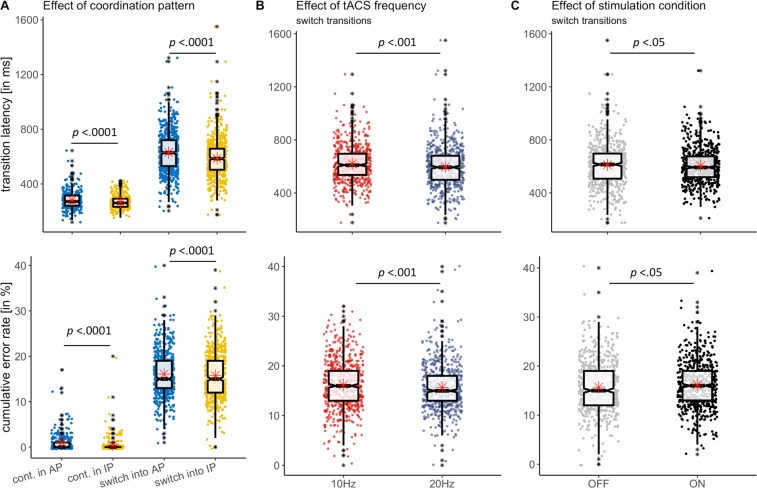
Single trial scatter and boxplots for cumulative error rate (in % of tapping/trial, LOWER PANEL) and transition latency (in ms, UPPER PANEL). (**A**) Effect of coordination pattern (AP vs. IP). Overall, irrespective of stimulation frequency and condition, IP transitions were less erroneous and faster for both, continuation and switching transitions. (**B**) Effect of stimulation frequency (10 Hz vs. 20 Hz) on switching transitions here pooled over coordination pattern (IP, AP) and stimulation condition (ON and OFF). Switching transitions were less erroneous and faster in the beta tACS condition (20 Hz). (**C**) Effect of stimulation condition (stimulated vs. non-stimulated trials in this representation pooled over coordination pattern (IP, AP) and tACS frequency. Stimulated switching transitions (ON) were faster but more erroneous. Computed variables for boxplots: lower/upper whiskers represent smallest/largest observation greater than or equal to lower hinge ± 1.5 * inter-quartile range (IQR), lower/upper hinge reflects 25%/75% quantile, lower edge of notch = median −1.58 * IQR/sqrt(n), middle of notch reflects group median, red asterisks represent group mean.

Subsequent discriminant analysis revealed no discriminant function for factor COORDINATION PATTERN. Both, transition latency (*b* = −0.006) and error rate (*b* = −1.056) were expectedly lower during IP continuation as compared to AP continuation (group means: transition latency/IP = 264.06, transition latency/AP = 273.07, error rate/IP = 0.10, error rate/AP = 0.42, Fig. 4A).

Furthermore, a significant interaction of STIMULATION FREQUENCY and TIME progression in the experiment (Pillai’s trace = 0162, *F*(2, 580) = 4.78, p < 0.01) indicated a significant differentiation of the stimulation frequency on the continuation performance over time, showing stable transition latencies over time with both 10 Hz and 20 Hz tACS but a significant increase in error rate over time in the 20 Hz tACS condition while the opposite was true for the 10 Hz tACS condition.

### Multivariate Analysis of tACS effects on switching transitions

In the switch trial data subset, MANCOVA revealed a significant main effect of COORDINATION PATTERN (Pillai’s trace = 0.029, F(2, 1180) = 17.53, p < 0.0001, Fig. 4A), a significant influence of STIMULATION FREQUENCY (Pillai’s trace = 0.0096, F(2, 1180) = 5.72, p < 0.005), and STIMULATION CONDITION (Pillai’s trace = 0.0049, F(2, 1180) = 2.65, *p* = 0.05, overview given in Table 2). The subsequent discriminant analyses revealed a single discriminant function for STIMULATION CONDITION (ON vs. OFF). The coefficients of discrimination showed that stimulated trials had lower transition latency (*b* = −0.005), while cumulative error rate increased (*b* = 0.224) compared to non-stimulated trials (group means: transition latency/stimulation OFF = 611.65, transition latency/stimulation ON = 604.93, error rate/stimulation OFF = 15.70, error rate/stimulation ON = 16.15, Fig. 4C).

**Table 2:** MANCOVA results for switching trials.

Multivariate Effects	Pillai’s trace	Approx *F* (*df*, Error *df*)	Pr(>F)
COORDINATION PATTERN	0.0289	17.53 (2, 1180)	0.00000003***
STIMULATION FREQUENCY	0.0096	5.72 (2, 1180)	0.003**
STIMULATION CONDITION	0.0049	2.92 (2, 1180)	0.05*
TIME centred	0.0045	2.65 (2, 1180)	0.07
TARGET FREQUENCY centred	0.0226	13.66 (2, 1180)	0.000001***
COORDINATION PATTERN*STIMULATION FREQUENCY	0.0004	0.21 (2, 1180)	0.8
COORDINATION PATTERN*STIMULATION CONDITION	0.0031	1.81 (2, 1180)	0.2
STIMULATION FREQUENCY*STIMULATION CONDITION	0.0009	0.55 (2, 1180)	0.6
COORDINATION PATTERN*TIME centred	0.0039	2.31 (2, 1180)	0.1
STIMULATION FREQUENCY*TIME centred	0.0024	1.46 (2, 1180)	0.2
STIMULATION CONDITION*TIME centred	0.0003	0.21 (2, 1180)	0.8
COORDINATION PATTERN*STIMULATION FREQUENCY*STIMULATION CONDITION	0.0003	0.16 (2, 1180)	0.9
COORDINATION PATTERN*STIMULATION FREQUENCY*TIME centred	0.0001	0.03 (2, 1180)	0.9
COORDINATION PATTERN* STIMULATION CONDITION*TIME centred	0.0001	0.07 (2, 1180)	0.9
STIMULATION FREQUENCY*STIMULATION CONDITION*TIME centred	0.0010	0.61 (2, 1180)	0.5
COORDINATION PATTERN*STIMULATION FREQUENCY*STIMULATION CONDITION*TIME centred	0.0046	2.73 (2, 1180)	0.07
Residuals	1181		
Residual standard errors	128.209		
	4.33		
Error: subject level	Pillai’s trace	approx *F* (*df*, Error *df*)	
TARGET FREQUENCY centred	0.1447	1.35 (2, 16)	
Residuals	17		

Type I sums of squares given. Asterisks indicate level of significance at ***p < 0.0001, **p < 0.01, *p < 0.05.

In all other cases, there was no discrimination of outcome variables for factor levels. Factor COORDINATION PATTERN showed that switches into IP coordination led to comparatively lower transition latency (*b* = −0.008) and lower cumulative error rate (*b* = −0.021; group means: transLatency/IP = 586.62, transLatency/AP = 629.97, errRate/IP = 15.75, errRate/AP = 15.09). Factor STIMULATION FREQUENCY revealed that 20 Hz tACS led in general to lower transition latency in switch trials (*b* = −0.007) and lower error rate (*b* = −0.097) compared to 10 Hz tACS (group means: transition latency/10 Hz = 620.17, transition latency/20 Hz = 596.41, error rate/10 Hz = 16.17, error rate 20 Hz = 15.57, Fig. 4B).

Furthermore, the tapping frequency had also a significant influence on the performance in switching trials (TARGET FREQUENCY, Pillai’s trace = 0.023, F(2, 1180) = 13.66, p < 0.0001), showing a minor increase of transition latency with increasing target frequency and a more pronounced increase in error rate with increasing target frequency.

### Analysis of tACS effects on Switching Costs

We furthermore analysed the effect of 10 Hz and 20 Hz stimulation in comparison to non-stimulated trials pooled over both tACS conditions (tACS CONDITION 10 Hz, 20 Hz versus OFF) separately for error rate and transition latency based switching costs (results summarized in Table 3).

**Table 3:** Results of the LME models for switching cost.

Switching Costs	Wald statistics			Type III sums of squares test	
	Parameter estimates (SE)	t-value (df)	Pr(>|t|)	*F*-value (df)	Pr(>F)
**a) based on transition latency**					
Fixed effects					
COORDINATION PATTERN	[IP] −24.81 (12.34)	−2.01 (888.0)	0.045*	27.458 (1, 888.0)	0.000***
tACS CONDITION	[20 Hz] −7.10 (12.34)	−0.58 (888.0)	0.57	3.681 (2, 888.0)	0.03*
	[10 Hz] 9.46 (12.34)	0.77 (888.0)	0.44		
COORDINATION PATTERN* tACS CONDITION	[IP, 20 Hz] −23.92 (17.45)	−1.37 (888.0)	0.17	0.946 (2, 888.0)	0.39
	[IP, 10 Hz] −13.65 (17.45)	−0.78 (888.0)	0.43		
Random effects	Variance component (SD)				
Intercept|subject level	5687 (75.41)				ω^2^ = 0.35
Residual	11574 (107.58)				
**b) based on error rate**					
Fixed effects					
COORDINATION PATTERN	[IP] −0.03 (0.46)	−0.06 (888.0)	0.95	0.512 (1, 888.0)	0.48
tACS CONDITION	[20 Hz] 0.09 (0.46)	0.18 (888.0)	0.85	4.314 (2, 888.0)	0.01*
	[10 Hz] 0.70 (0.46)	1.52 (888.0)	0.13		
COORDINATION PATTERN* tACS CONDITION	[IP, 20 Hz] 0.23 (0.65)	0.36 (888.0)	0.72	0.212 (2, 888.0)	0.81
	[IP, 10 Hz] 0.43 (0.65)	0.65 (888.0)	0.52		
Random effects	Variance component (SD)				ω^2^ = 0.31
Intercept|subject level	7.16 (2.68)				
Residual	16.27 (4.033)				

Parameter estimates are given for factor levels given in squared brackets with respect to AP COORDINATION PATTERN and OFF tACS CONDITION as reference categories. Asterisks indicate level of significance at ***p < 0.0001, **p < 0.01, *p < 0.05 estimated for LME models with Satterthwaite approximation. For both models the number of observations was 912 for 19 groups, i.e. subject.

**Figure 5:**
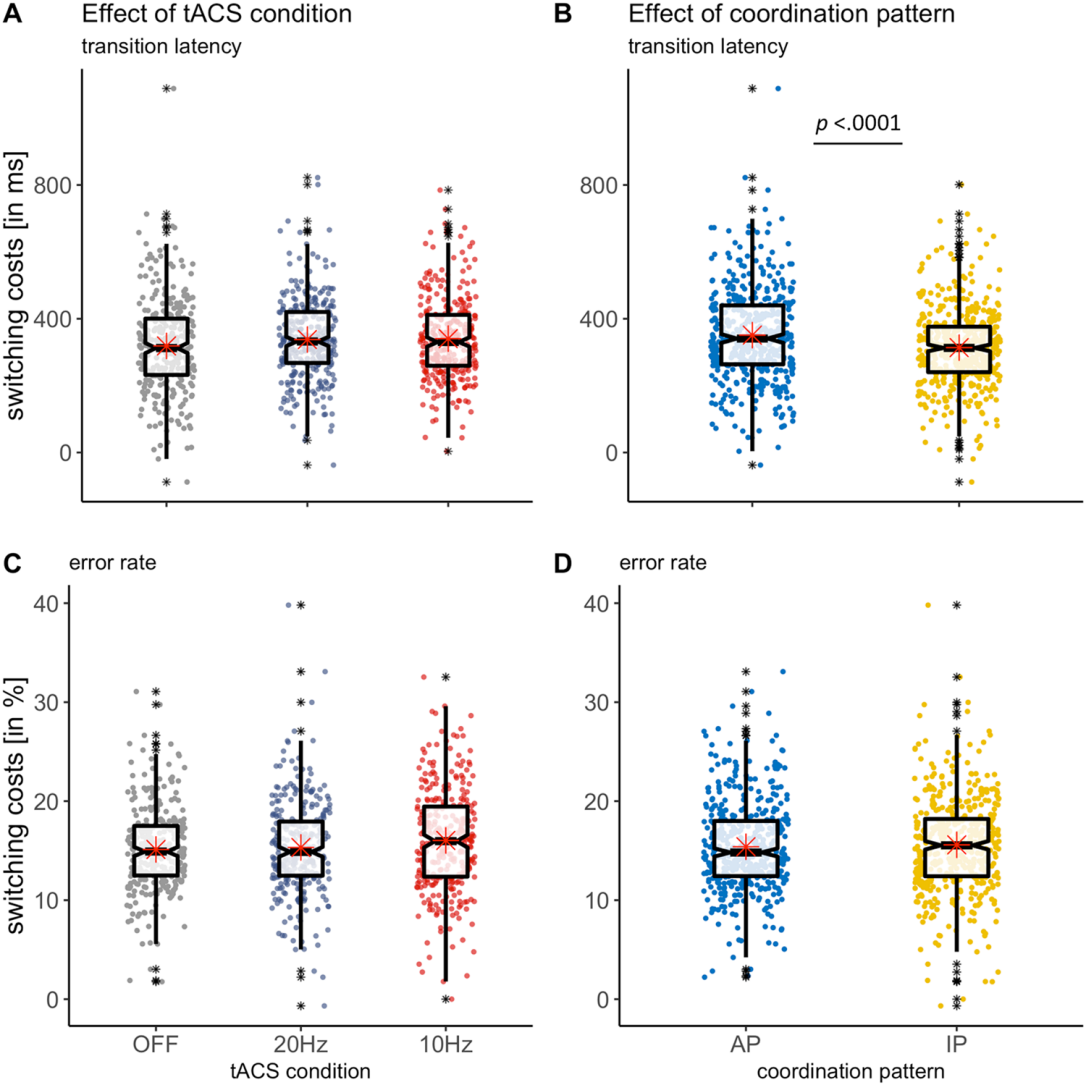
Switching costs, i.e. relative disadvantage of switching transitions compared to continuation transitions. (**A**) Transition latency based switching costs were modulated by tACS condition (p < 0.05) with a non-significant trend for highest switching costs under 10 Hz and lowest switching costs under 20 Hz. (**B**) Transition latency related switching were lower for the IP coordination pattern (p < 0.0001). (**C**) Main effect of tACS condition (p < 0.05) on error rate based switching costs. (**D**) No effect of coordination pattern on error rate based switching costs.

Transition latency related switching costs showed a main effect of COORDINATION PATTERN (*p* < 0.0001) with overall higher switching costs for transitions into AP, i.e. the more difficult coordination pattern, of approximately 25 ms as revealed by the parameter estimates (Fig. 5). Furthermore, a significant main effect of tACS CONDITION (*p* < 0.05) suggested overall highest switching costs for the 10 Hz condition, which did however not reveal significant pairwise differences to the 20 Hz or non-stimulated condition (all p > 0.1). The error rate based switching costs were equally modulated by tACS CONDITION (*p* < 0.05, Fig. 5) but without significant contrasts between the different conditions (all p > 0.1).

### Analysis of non-stimulated trials alone to investigate carry-over effect of stimulation

Driven by the aforementioned findings, namely (1.) the interaction effect of stimulation frequency and time on continuation trials (more errors in continuation trials in the 20 Hz session over time) and (2.), the discrimination of the stimulation condition effect on switch trials (faster but more errors when stimulation was on), we performed a separate analysis of non-stimulated trials to further investigate a potential carry-over effect of the event-related short tACS trains on non-stimulated trials (overview of statistical results given in Table 4 for transition latency and in Table 5 for cumulative error rate).

**Table 4:** LME model results for transition latency in offline stimulation condition.

Transition latency	Wald statistics			Type III sums of squares test	
	Parameter estimates (SE)	t-value (df)	Pr(>|t|)	*F*-value (df)	Pr(>F)
**a) continuation transitions**					
Fixed effects					
STIMULATION FREQUENCY	[20 Hz] 1.51 (4.2)	0.36 (586.3)	0.72	0.13 (1, 586.3)	0.72
TIME centred	1.82 (3.0)	0.62 (585.2)	0.54	0.45 (1, 585.3)	0.5
TARGET FREQUENCY centred	−57.70 (6.7)	−8.67 (103.2)	0.000***	75.06 (1, 103.2)	0.000***
STIMULATION FREQUENCY*TIME centred	[20 Hz] −2.61 (4.2)	−0.63 (585.2)	0.53	0.39 (1, 585.2)	0.53
Random effects	Variance component (SD)				ω^2^ = 0.26
Intercept|subject level	254 (15.94)				
Residual	2655 (51.53)				
**b) switching transitions**					
Fixed effects					
STIMULATION FREQUENCY	[20 Hz] −25.89 (11.97)	−2.16 (585.8)	0.03*	4.67 (1, 585.8)	0.03*
TIME centred	0.20 (8.46)	0.02 (585.0)	0.98	0.87 (1, 585.0)	0.35
TARGET FREQUENCY centred	2.11 (21.31)	0.10 (225.5)	0.92	0.01 (1, 225.5)	0.92
STIMULATION FREQUENCY*TIME centred	[20 Hz] −17.45 (11.96)	−1.46 (585.0)	0.15	2.13 (1, 585.0)	0.15
Random effects	Variance component (SD)				ω^2^ = 0.21
Intercept|subject level	4883 (69.88)				
Residual	21734 (147.42)				

Parameter estimates are given for factor levels given in squared brackets with respect to 10 Hz STIMULATION FREQUENCY as reference cate gories. Asterisks indicate level of significance at ***p < 0.0001, **p < 0.01, *p < 0.05 estimated for LME models with Satterthwaite approximation. For both models the number of observations was 912 for 19 groups, i.e. subject.

**Table 5:** LME model results for cumulative error rate in offline stimulation condition.

Cumulative error rate	Wald statistics			Type III sums of squares test	
	Parameter estimates (SE)	t-value (df)	Pr(>|t|)	*F*-value (df)	Pr(>F)
**a) continuation transitions**					
Fixed effects					
STIMULATION FREQUENCY	[20 Hz] 0.12 (0.1)	0.84 (586.4)	0.4	0.70 (1, 586.4)	0.40
TIME centred	−0.07 (0.1)	−0.73 (585.4)	0.46	3.46 (1, 585.4)	0.06
TARGET FREQUENCY centred	0.25 (0.2)	1.12 (101.4)	0.26	1.26 (1, 101.4)	0.26
STIMULATION FREQUENCY*TIME centred	[20 Hz] 0.41 (0.1)	2.89 (585.4)	0.003**	8.38 (1, 585.4)	0.004**
Random effects	Variance component (SD)				ω^2^ = 12
Intercept|subject level	0.28 (0.53)				
Residual	2.98 (1.73)				
**b) switching transitions**					
Fixed effects					
STIMULATION FREQUENCY	[20 Hz] −0.34 (0.4)	0.903 (586.2)-	0.37	0.82 (1, 586.2)	0.37
TIME centred	0.06 (0.3)	0.235 (585.4)	0.81	0.40 (1, 585.4)	0.53
TARGET FREQUENCY centred	1.71 (0.7)	2.614 (204.6)	0.01**	6.83 (1, 204.6)	0.01*
STIMULATION FREQUENCY*TIME centred	[20 Hz] 0.11 (0.4)	0.296 (585.4)	0.77	0.09 (1, 585.4)	0.77
Random effects	Variance component (SD)				ω^2^ = 0.21
Intercept|subject level	4.22 (2.05)				
Residual	21.09 (4.59)				

Parameter estimates are given for factor levels given in squared brackets with respect to 10 Hz STIMULATION FREQUENCY as reference categories. Asterisks indicate level of significance at ***p < 0.0001, **p < 0.01, *p < 0.05 estimated for LME models with Satterthwaite approximation. For both models the number of observations was 912 for 19 groups, i.e. subject.

**Figure 6:**
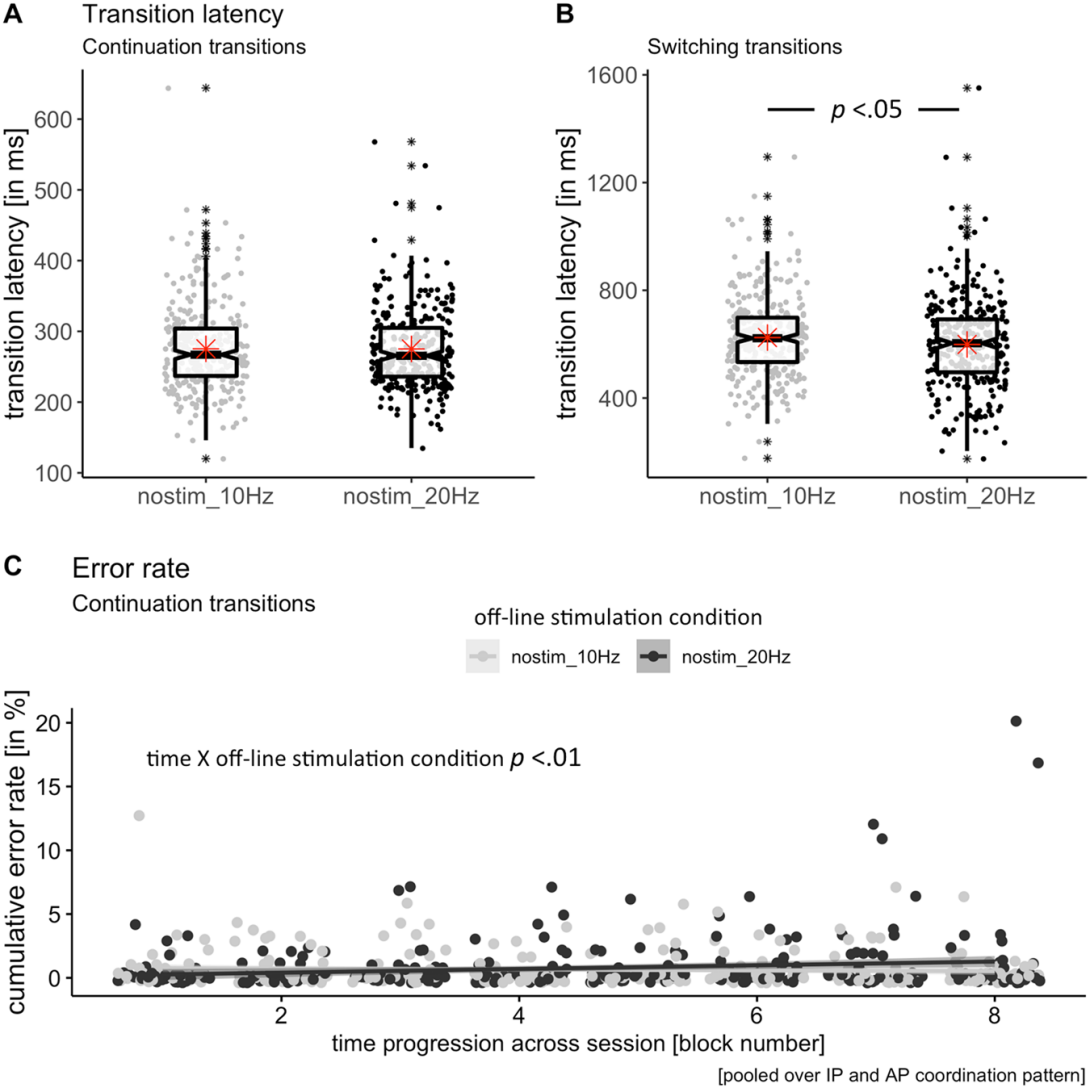
Boxplots and single trials scatter plots showing the effect of offline stimulation condition on transition latency (**A**) in continuation and (**B**) in switching transitions. Switching transitions in the offline 20 Hz condition were comparably faster than those in the offline 10 Hz condition. Computed variables for boxplots: lower/upper whiskers represents smallest/largest observation greater than or equal to lower hinge ± 1.5 * inter-quartile range (IQR), lower/upper hinge reflects 25%/75% quantile, lower edge of notch = median − 1.58 * IQR/sqrt(n), middle of notch reflects group median, red asterisks represent group mean. (**C**) Diverging effect of time progression in the two different offline stimulation conditions on cumulative error rate. While a decrease of error rate was found for the offline 10 Hz condition, an increase in error rate was found in the offline 20 Hz condition over time (p < 0.01) in continuation trials. Regression line with 95%CI presented for each group. Offline 10 Hz tACS is depicted in grey, offline 20 Hz tACS depicted in black in non-stimulated trials pooled over coordination pattern (IP, AP).

This analysis revealed that the 20 Hz condition led to shorter transition latencies in switching transitions (parameter estimates −25.89, *p* < 0.05, Fig. 6A) in comparison to the 10 Hz condition when corrected for individual tapping frequency. This main effect of STIMULATION FRQUENCY on transition latency was absent for the non-stimulated continuation transitions.

For the cumulative error rate time progression (TIME centred) across the experiment showed a numerically small but statistically significant interaction with the STIMULATION FREQUENCY for continuation transitions (p < 0.01, Fig. 6C). Parameter estimates of the Wald statistics for this model revealed that error rate tended to increase in continuation transitions over time in the 20 Hz condition (0.41, p < 0.01), while the opposite trend was visible in the 10 Hz condition (parameter estimate for 20 Hz as reference category −0.41, p < 0.01).

## Discussion

Based on the relevance of sensorimotor alpha and beta rhythms for neural inhibitory mechanisms^21–25^ and the hypothesis of more effective inter-areal information transfer through synchronized neural oscillations^45,46^, we explored the behavioural effects of bihemispheric in-phase tACS with 10 Hz versus 20 Hz stimulation frequency on a coordinative challenging bimanual task-set switching paradigm.

Our analysis of the tACS effect on bimanual task-set switching behaviour revealed four key findings: (1.) A main effect of stimulation condition (stimulation ON versus OFF) showing that online tACS in either frequency led to faster but more erroneous switching transitions; (2.) a significant influence of stimulation frequency (10 versus 20 Hz) showing that 10 Hz in-phase stimulation had a detrimental effect on switching transitions in both domains, i.e. error rate and speed; (3.) no change of the relative disadvantage of switching compared to continuation (switching costs) by tACS, and (4.) a differentiation of stimulation frequency in non-stimulated trials following the stimulation trains indicating offline stimulation effects.

Irrespective of the stimulation condition or frequency, we observed an expected pattern of increased time needed to perform a transition between two different coordination patterns, i.e. the switching costs reflecting the relative disadvantage of switching between two different task sets versus the continuation of the same task set. These observations are well in accordance with previous work using a comparable motor paradigm to investigate intentional task-set switching^43^ and follow the predictions of the Schöner-Kelso theory of intentional behavioural change^47^. Furthermore, our data confirmed an increase in transition latency when transitions were performed from the more stable IP into the less stable AP coordination mode as compared to the reversed direction, reflecting the expected asymmetry of switching costs^40–42^. The same pattern was observable in the outcome reflecting the quality of performance, i.e. the error rate, which was generally higher in switching transitions and in transitions into the AP coordination mode.

Albeit causal evidence regarding the exact role of spectral information in interhemispheric coupling during complex bimanual movements is lacking, which would allow us to fully elucidate our findings, a potential explanation for the present observations may be developed in the light of available knowledge regarding the control of bimanual coordination and the hypotheses about the mode of action of tACS.

The bifocal 10 Hz and 20 Hz tACS with zero phase-lag (in-phase) between the motor regions of left and right hemisphere used in the present study may have potentially led to a synchronization of long-range interhemispheric sensorimotor oscillations. This idea has been suggested by previous tACS studies targeting interregional information transfer between homologous visual^34^, sensorimotor^48,49^, or between frontal and parietal^38,39^ regions with concurrent tACS and fMRI or EEG. Only recently however, dual-site TMS work has generated direct evidence for the relevance of phase synchronicity of rolandic alpha oscillations for the inhibitory interaction between homologous primary motor cortices^50^. This study revealed that interhemispheric inhibition was most pronounced with in-phase alpha oscillation at the time point of the negative peak but leaving open the question for their behavioural relevance. Furthermore, a recent computational modelling study has shown elegantly that it is possible to achieve two spatially differentiated electrical fields to target inter-regional phase synchronization with the centre-ring montage used in our study^51^ while montages consisting of two or three patch electrodes lead to a widespread distribution of the electrical field between the electrodes and beyond^52,53^, which clearly limits inferences about the effects of the applied electrical stimulation on specific regions or their interaction.

Long-range interhemispheric sensorimotor synchronization has been shown to increase in the alpha and beta band with higher coordinative effort and task demand for various different uni- and bimanual dexterous activities^18,54,55^. In particular the interhemispheric beta band connectivity seems to be modulated by dynamic features of manual movements^18,56,57^. Noteworthy, irrespective of stimulation frequency, our event-related design led to a clear distinction between stimulated and non-stimulated switching transitions with a dissociation of the two outcome measures: While under online stimulation with both 10 Hz and 20 Hz tACS, switching transitions were generally faster, they showed an overall higher error rate than trials which were not actively stimulated. This may be indicative for the relevance of stronger interhemispheric coupling for movement dynamics resulting in higher speed of bimanual coordination, i.e. faster transition latency in our study in a broader sensorimotor frequency range.

Contrary to our hypothesis, the two different stimulation frequencies had no specific effect on the direction of switching transitions, i.e. the relative disadvantage for transitions from IP into AP remained present irrespective of stimulation condition. This may be a result of the present approach of using a generic stimulation frequency within the alpha and beta band instead of individually adjusting the stimulation frequency. Another limitation may be the comparably low stimulation intensity used here, which may have been ineffective in critically modulating the local intrinsic neural mechanisms to impact specifically on defined aspects of bimanual coordination. Alpha and beta frequency bands show a differentiation in their topographic and temporal features with beta modulation being spatially more focussed and earlier in occurrence than motor-related alpha modulation^27,58,59^. It is therefore possible that the stimulation parameters used in our study were not sensitive enough in terms of frequency or timing to distinguish between the local control mechanisms of transitions between the two coordination modes (IP/AP). The resulting beta increase may have been too coarse in time and have overruled the fine-tuned de- and re-synchronization dynamics required for the two switching directions. Elevated beta synchronization within the primary motor cortex is known to occur during tonic muscle activation^60^ or during voluntary suppression of movements^61–63^. These observations have motivated the idea that motor cortical beta synchrony is an important means to assure steady motor output and counteract unintended movement initiation^25^. The present finding of increased switching transition latency under 20 Hz tACS is clearly contrasting this hypothesis and is inconsistent with previous work in which 20 Hz tACS has led to movement slowing^31,64,65^. Besides the fact that none of the previous work has examined stimulation effects on bimanual task-set switching, one possible explanation may be the specific requirement of the steady production of manual behaviour with interleaved transitions in our study in contrast to single discrete events of reactions used in the previous work. Additionally, the stimulation parameters (electrode montage, stimulation duration) clearly differentiate the present from previous work.

Nevertheless, during stimulation, the switching transitions not only became faster but also more erroneous, independent of stimulation frequency. One simple explanation may be that an overall higher tapping speed during stimulation may have happened at the cost of more erroneous behaviour.

On a more speculative note, the flexible motor control required for the correct switching transition into the new coordination mode may have been hampered as a consequence of our tACS intervention. In particular, the specific asymmetric influence of the dominant onto the non-dominant hemisphere in bimanual control^66^ may be perturbed by an artificial up-regulation of the synchronization between both hemispheres. It may be the case that a high degree of connectivity achieved through interhemispheric synchronization, particularly under 10 Hz tACS (during which the negative effects were strongest), is detrimental for flexible control of complex bimanual actions. Support for this hypothesis is provided by findings in participants of advanced age in whom high-density EEG data has shown a strong inhibitory coupling between homologous primary motor areas (from left onto right primary motor cortex) during incongruent bimanual movements which was absent in young controls^67^.

We observed a numerically small but nonetheless clear change of performance over time in continuation transitions although these were not actively stimulated. Since our focus was set on the switching transitions, we chose not to stimulate continuation transitions for feasibility reasons to limit the overall tACS dosage, taking into account that our interpretation is limited with respect to potential effects of tACS on continuation transitions. Of note, time progression over the experiment had no significant influence on the performance during switching transitions. We therefore added an additional analysis of non-stimulated trials to further investigate a potential carry-over effect from stimulated to non-stimulated trials. This analysis revealed a small increase in cumulative error rate of continuation transitions over time within the 20 Hz session, while error rates declined throughout the 10 Hz session. No such interaction effect of tACS frequency and time progression was evident for the temporal feature of performance, i.e. the transition latency in continuation trials.

We suggest that the gradual increase in error rate under 20 Hz is indicative of differential offline effects of the two tACS frequencies, which has been shown previously for several minutes long continuous tACS applications^65^. Although still no consistent information exists regarding the exact parameters that cause enduring effects of tACS beyond the duration of the stimulation^68^, evidence accumulates that after-effects depend on the length of the stimulation train. In this regard, lasting effects on endogenous signal strength (power increase) in the stimulation frequency have been shown for intermittent stimulation of 8 seconds but not 3 seconds^69^ or 1 second^70^ duration. The nature of our task itself, i.e. the enduring fast rhythmic tapping in the continuation transitions, may be a cause for changes of the typical movement-related spectral dynamics^71^. Amplitude characteristics in the beta band merge with increasing performance speed, impeding the differentiation between de- and re-synchronization^20,72^. This endogenous effect may have been amplified by a latent effect of 20 Hz tACS, leading to the observed imprecision in continued rhythmic tapping. We therefore interpret our findings of increased error rate in the non-stimulated continuation trials in the beta session as an indication for true after-effects of the tACS induced up-regulation of beta synchronization.

A potential after-effect may hence not only depend on the actual duration of the stimulation train but also on the frequency of the external oscillator. For the higher frequency beta stimulation used here, this could imply that a short stimulation train of 5 seconds might have been sufficient to lead to so-called entrainment-echos^73^ or true after-effects. Since we observed an increase of the effect over time within the experimental session, we propose that these entrainment effects accumulated with the duration of the experiment and increasing number of stimulation trains. Importantly, since this was not the case for the 10 Hz condition it contradicts the idea of a direct effect on the harmonics, i.e. 10 and 20 Hz, of the stimulation frequency. However, it needs to be taken into account that we have not used a specific blinding procedure in our study, which leaves a chance that the participants have recognized the difference between the tACS conditions despite comparable levels of perceived discomfort. Future work needs to unravel the exact underlying neural mechanism for these behavioural findings and clarify whether for example a rebound phenomenon or a lasting synchronization is caused by the intermittent beta stimulation.

## Conclusion

Bifocal in-phase tACS with 10 Hz and 20 Hz leads to beneficial online effects on movement speed at the price of more errors in bimanual task-set switching. While beta tACS seems to be more efficient in speeding up task-set switching it also has a less detrimental online effect on the error rate during switching than alpha tACS. These results may be explained by an increased online in-phase synchronization of long-range interhemispheric sensorimotor oscillations, which potentially interferes with the hemispheric information flow and the top-down control required for flexible control of complex bimanual actions.

Furthermore, we argue that different stimulation frequencies lead to distinct offline effects, which potentially accumulate over time and therefore need to be taken into account when evaluating subsequent performance.

## Material and Methods

### Participants

The behavioural effect of modulating interhemispheric interactions was causally tested with alpha (10 Hz) versus beta (20 Hz) tACS in a double-blind cross-over design. Twenty right-handed^74^ (91.21 ± 22.45%) healthy volunteers (age range 20–26 years, average 23.04 ± 1.42 years, 10 female) were included in this study. Standard screening verified that none of the participants presented with contraindications regarding non-invasive brain stimulation^75,76^, was playing piano or proficient in 10-finger type writing. In accordance with the declaration of Helsinki (2008), all procedures were carried out as approved by the ethical committee of the University Medical Centre of the KU Leuven (Protocol No. 57103) and written informed consent was obtained from all participants.

### Behavioural paradigm

We made use of a well-described experimental movement paradigm with two patterns of differing stability: the in-phase or simultaneous (IP, high level of stability) and anti-phase or alternating (AP, lower level of stability) movement of two fingers of both hands and the externally cued intentional transition between these patterns e.g.^40–42^. The participants performed the IP-/AP tapping on a custom-made keyboard with four input keys (1000 Hz sampling rate). Individual tapping frequency was auditory paced through headphones during the complete experiment. Visual target cues indicated which movement pattern to perform. In an initial training period prior to each of the two experimental sessions, participants were first familiarized with the stimulus-response mapping and then practised the task. Individual target frequency was set to 90% of the frequency of comfortable AP performance before the unintended transition into the IP mode occurred spontaneously. Re-adjustment of the individual performance frequency was implemented in the second session because a general performance improvement was expected due to intensive practice within the first session.

Eight blocks of approximately 5 min. length were performed.

Visual and auditory stimuli of the behavioural paradigm were programmed in LabVIEW 2014 (National Instruments, Austin/TX, USA). A white fixation cross was presented against a black background on a computer monitor for the duration of the experiment. Each trial began with the presentation of an imperative cue, which remained on the screen for 800 ms. Colour coding informed the participant about the type of movement (continuation vs. transition). Cues indicating a different movement pattern at start or after a pause, were presented in green, cues indicating continuation of an on-going movement pattern were presented in white. In 50% of the trials, a transition cue was presented, in 40% of the trials a continuation cue was presented, 10% of the trials were ‘pause’ trials to avoid muscle fatigue during continuous tapping. The stimulus onset asynchrony (SOA; cue to cue interval) varied between 5 and 8 s in a random distribution, with an average of 6.3 s. The six conditions (IP continuation, AP continuation, transition into IP initiated with the right/left hand, transition into AP initiated with right/left hand) were presented in a pseudorandom sequence that included no more than three consecutive trials of the same condition. One block lasted approximately 5 minutes and consisted of 50 trials including all four conditions as well as ‘start’ trials at the beginning and after breaks (‘pause’ trials). Eight blocks were performed with 1–2 minutes breaks in between. Including preparation of electrode montage, familiarization and training, each experimental session was of approximately 90 minutes in duration.

### Transcranial alternating current stimulation (tACS)

Transcranial electrical stimulation was applied using an AC/DC-stimulator (NeuroConn, Ilmenau, Germany) with peak amplitude of 500 µA (1000 µA peak-to-peak amplitude). The participants attended two sessions with alpha (10 Hz) or beta (20 Hz) tACS counterbalanced across sessions in pseudo-randomized order. A minimum inter-session interval of 48 hours was chosen to avoid potential carry-over effects^77,78^.

Within each session, intermittent tACS was applied in an event-related fashion distributed randomly over 50% of the switch transition trials while continuation transitions were not stimulated. We followed this approach in order to focus on switching transitions and achieve a sufficient number of trials to compare the different stimulation conditions by keeping within the safety limits for total tACS applied^79^. Based on the assumption of an online effect mainly during tACS^36,80–82^, the stimulation commenced 1000 ms before visual cue onset and lasted for a total of 5000 ms in order to fully cover the actual transition phase with the stimulation train (Fig. 1B), including ramping in and out phase over 1 cycle. Between the end of the previous and the start of the next stimulation train, a minimum interval of 5000 ms elapsed. Over one experimental session all participants received a total duration of approximately 11 min. of tACS, complying with the limits given in currently available safety recommendations^76,79^.

We chose a bifocal centre-ring electrode montage designed to better focalize the electrical field over the primary motor area to target interhemispheric interactions^83^. Centre electrodes were positioned over the left and right primary motor cortex, identified by the electrode position of C3/C4 of the international 10–20 EEG system^84^, each with a surface area of 9 cm^2^, diameter 3.4 cm. For the centre-ring montage the ‘return’ electrode consisted of ring-shaped patch electrodes surrounding the centre electrode with a 2.05 cm distance between electrode borders (individual surface area 35 cm^2^, inner diameter 7.5 cm, outer diameter 10 cm, Fig. 2).

By using Y-connector cables, a 0° phase difference was achieved between centre electrodes and between outer ring electrodes, respectively. Conductive gel was applied on the electrodes to assure that compound impedance was kept below 10 kΩ throughout the experiment. The electrical field of the bifocal centre-ring montage was simulated for an example participant based on the SimNIBS 2 pipeline (www.simnibs.org) and a finite element head model derived from individual MRI data of the same participant^85,86^. All electrodes were modelled as 1 mm thick rubber layers (conductivity 0.1 S/m) with 1 mm thick layers of conductive gel underneath (conductivity of 14 S/m, estimated based on the concentration of sodium in the Onestep Cleargel, as stated by the manufacturer). The positions of the connectors were explicitly modelled (modelling procedure described in detail in^86^).

### Behavioural outcome parameters

Performance in the bimanual task-set switching paradigm was described with three outcome parameters to characterize performance quality and the temporal features in the transition phases: (1.) error rate and (2.) transition latency, and (3.) switching costs reflecting the relative disadvantage of switching between coordination patterns compared to continuation of the same pattern.

#### Error rate (errRate)

We defined performance error as the cumulative error rate of tapping within the time window of interest (in % of total taps in the time window) between cue onset and the following cue. A trial showing 100% of erroneous tapping was considered a failed transition. These trials were not considered for the calculation of the transition latency.

#### Transition Latency (tL)

The transition latency (*tL*) was defined as the time delay between cue onset and response, i.e. the first occurrence of the correct coordination pattern indicated by the cue. To be valid, correct responses to visual cues had to have a latency ≥100 ms and <2000 ms, in order to allow for true visual processing of the cue and visuomotor integration for the correct response^87–89^.

#### Switching Costs (SC)

Switching costs define the relative disadvantage of performing a switch in task-set in comparison to repeating the same task. For the study here, switching costs (*SC*) were therefore operationalized on the one hand based on the temporal features (transition latency) as

$$S{C}_{tL}=t{L}_{switch}-t{L}_{continuation}$$

where *tL* is the transition latency for switch and continuation transitions;

and on the other hand based on the quality of the performance (the error rate) with

$$S{C}_{er}=er{r}_{switch}-er{r}_{continuation}$$

where *err* is the cumulative error rate of tapping in the time window of interest [cue onset :2000 ms post cue] for switch and continuation transitions, respectively. For this analysis, the same criteria for exclusion of trials were applied as described above for error rate and transition latency.

All offline processing of kinematic data was performed using custom scripts (MATLAB 2015b, Mathworks, Natick, MA, USA).

### Evaluation of subjective level of discomfort caused by tACS and self-perceived level of fatigue

Level of discomfort was assessed according to a Visual Analogue Scale (VAS) of 100 mm length without numerical indication of which conversion into numbers (0–10) was only done afterwards^90^. The VAS-Discomfort (extremes constituted of “absolutely no discomfort/pain” and “worst discomfort/pain ever”) was only assessed after each session. Participants self-evaluated their subjective level of neurosensory discomfort caused by tACS by placing a line perpendicular to the VAS-line at the point that represents their perceived intensity of “discomfort/pain”.

Furthermore, participants evaluated their perceived level of fatigue with a VAS (extremes constituted of ‘absolutely not tired’ and ‘maximally tired/exhausted’) at the beginning and end of each experimental session as described for discomfort.

### Statistical analysis

Intersession differences of experimental and participant characteristics, i.e. training duration, performance frequency and impedance ranges were analysed with a one-way analysis of variance (ANOVA).

The effect of the stimulation intervention on bimanual task-set switching behaviour was operationalized as cumulative error rate and transition latency and was analysed with a multivariate analysis of covariance (MANCOVA) for continuation trials and switch trials separately given their difference in distribution, with a significantly wider spread in the switching transitions for both dependent variables. As independent variables, COORDINATION PATTERN (IP, AP), STIMULATION FREQUENCY (10 Hz, 20 Hz), and STIMULATION CONDITION (ON, OFF) were modelled as factors, TIME progression over the experiment was modelled as covariate (block number, centred) for both, continuation and switching transitions. Although continuation transitions were not stimulated, we also included a variable called STIMULATION FREQUENCY in the model in order to analyse a systematic within session effect of the 10 Hz versus the 20 Hz condition. This means, if a carry-over of the stimulation effect on the non-stimulated trials was present, we were interested whether this may also have affected the continuation transitions. To correct for individual differences in tapping frequency, TARGET FREQUENCY (centred) was modelled as covariate. Pillai’s trace is given as test statistics for MANCOVA results in addition to transformed degrees of freedom for the F-ratio of the two dependent variables (approx *F*, *df*, Error *df*). In order to define the direction of the effects, MANCOVA was followed up with discriminant function analysis (DFA) for significant predictors. Coefficients of linear discriminants are given for DFA results.

The effects of coordination pattern and stimulation condition on transition latency and error based switching costs were modelled with separate LME models for the two switching cost modalities. For this analysis, non-stimulated trials were pooled across stimulation frequencies and tACS CONDITION was modelled as a three level (OFF, 10 Hz, 20 Hz) fixed factor, together with COORDINATION PATTERN (IP, AP) and their interaction.

For the analysis of non-stimulated trials, IP and AP patterns were pooled, as coordination pattern had not shown to be a significant modulator of STIMULATION CONDITION.

Separate linear mixed effects models were fit for transitions latency and error rate of continuation and switch trials. The effect of time progression within the experiment was modelled as linear polynomial (TIME centred) in order to identify a potential carry-over effect of tACS from the stimulated on the non-stimulated trials over time. The design variables STIMULATION FREQUENCY (10 Hz, 20 Hz) and TIME progression and their interaction were modelled as fixed factors. To control for individual differences in tapping frequency, TARGET FREQUENCY (centred) was included in the LME models. For all LME models, random intercepts were modelled on SUBJECT level using restricted maximum likelihood criteria (REML) and Satterthwaite approximation for significance testing.

Results for LME models are given as Type III sums of squares for sequentially fitted fixed effects (*F*, df, Pr(>F)), Wald statistics for marginal parameter estimates (*t*, df, *p*, 95% CI), as well as variance component estimates for random effects and respective standard deviations (variance, SD). Descriptive statistics were given as average ± standard deviation unless indicated differently.

All statistical analyses were performed using the software package R for Statistical Computing, version 3.4.1^91^ for x86_64-apple-darwin 15.6.0 (64-bit) running under OS X 10.11.6 using R packages MASS version 7.3–47, car version 2.1–5 for MANCOVA and DFA, and packages nlme version 3.1–131^92^, lme4 version 1.1-18-1^93^, lmerTest version 3.0-1^94^, version 3.0.0^95^ for building LME models with Satterthwaite approximation, multcomp version 1.4–8^96^ for posthoc comparison, and ggplot2 version 3.0.0^95^ for visual representation of results.
